# How to Treat Alveolar Abscess

**Published:** 1882-05

**Authors:** L. D. Carpenter

**Affiliations:** Atlanta, Ga.


### ARTICLE V.

### *How to Treat Alveolar Abscess.*

BY DR. L. D. CARPENTER, ATLANTA, GA.

The subject of alveolar abscess is one fraught with great interest to the dental profession. Much has been written on the subject. Yet as many are in the dark as to the best mode of treatment, I give you an item in my own practice.

Some twenty-five years ago, in the youth of my professional life, I was very much troubled and annoyed, aye, vexed, with the stubbornness of alveolar abscess, connected with dead teeth, in which the nerve had, from the inroads of decay, or the close proximity of a metallic filling become devitalized, abscess formed with a fistulous opening through the labial surface of the gum.

My style of practice at that time was, (and I believe it was the adopted rule by the profession) to excavate or open up the nerve cavity, remove therefrom all *debris* and decayed particles as perfectly as possible to the apex of the fangs, cleanse the nerve cavity nicely with tepid water and alcohol, then wind a shread of cotton around a nerve broach, dip in creosote, press the broach into the nerve cavity, and pump the cresote if possible, through into the sack at the end of the tooth and thence along the track of the abscess to the external opening on labial surface, then pack the nerve cavity with cotton, the first little pellet having been moistened with creosote—dismiss the patient to return again in one or two days, when the same treatment was repeated as before and patient dismissed to return again in two or three days.

These frequent visits were made for weeks, and perhaps sometimes for months, before I could feel satisfied that the abscess was cured, and it was safe to fill the tooth.

During this interval twenty-five or thirty sittings have been given the patient and about forty-five minutes of valuable time given to each visit; the tooth is filled, nerve and crown cavity, nicely, with gold, occupying about three hours; a charge is made of the nominal sum of five dollars; the operator straightens up his, then almost broken back, wipes his dimmed eyes and has a feeling of satisfaction come over him that he has done his every duty faithfully for himself and his patient, and is now ready to receive the thanks of an appreciative patron. Whilst thus musing, a sound greets his ear, a "still small voice" is heard, and these words like peals of thunder come rolling up before him: "Doctor, your charge is *outrageous*! why, Dr. So-and-so treated a tooth for Mrs. Spilkins for one solid year, filled it with gold from top to bottom, and only charged her *one dollar!* and took his pay in chickens at fifty-cents apiece! You are an impostor, Sir!"—Exit.

Thus endeth the first lesson.

In 1862, after taking account of stock, I found on hand: One wife, a house full of children and five dollars in Confederate money, with the buttons off the boy's jacket and a spool of white cotton, then worth ten dollars—(Fiction.)

With this view of the situation, and the dear ones around me begging for buttons, thread and more chicken, I decided to change my base of operations, erect new fortifications, enlist a new army, and attack the aforesaid class of teeth from an entirely new stand-point.

From that day to this, (20 years,) I have treated all teeth as herein described, after this wise: Having prepared and cleansed the nerve cavity nicely as possible, I force creosote or carbolic acid through the nerve canal into the abscess at the end of tooth, until I can see it come out freely at the opening on the labial surface; then I know the sack is broken, and the entire track to the fistulous opening is submerged with creosote. I then roll a small piece of soft gold foil, No. 4, around the point of a delicate "nerve plugger," dip it in creosote or carbolic acid, and pass it along the nerve canal to the foramen at the apex of root; the gold slips from the plugger and is packed, by gentle pressure, firmly to the walls and bottom of the cavity. Continue filling with gold until the nerve cavity is one-half or two-thirds full; then to prevent communication with the outside world, in the way of electricity, galvanism, or any other "ism," I fill the remainder of nerve cavity with any cement filling which will act as a non-conductor, and the remaining crown cavity I fill with gold. This is all done at the first or second sitting of the patient, with not more than one or two days intervening between appointments; and in a practice of twenty years, all teeth of this character, from the central incisor to the second bicuspid have been, in my hands, an almost universal success, without any regard to the time and alveolar abscess has been in existence.

In my opinion the sequel to this "whole business" is this; The nervous system is one vast circuit of telegraphic communication, with grand trunk and branch lines running in every direction. General distributing offices are established in various sections of the circuit, and branch offices at the end of every line, with the brain as the great home office or grand centre.

The nerve of a tooth is disturbed or interfered with, from some cause, and unable to perform its function; a dispatch is immediately sent to the nearest distributing office, and from thence to the home office; a detachment of extra laborers is immediately sent out to repair the break. When they arrive on the ground, and view the situation, they find a sack at the end of the tooth, the poisonous contents of which are oozing out, and have already destroyed the "osseons" walls on one side and passed out at an opening on the labial surface. The workmen clear away the rubbish and commence as best they can to lay a new foundation; all are working nicely, songs are being sung, and the wall are going up slowly, but securely, when methinks a slight scratching sound is heard in the tooth beyond them; presently a rushing noise is heard in the distance as of a mighty tornado, and here comes, through the foraman at the end of the root, alcohol, hot water, creosote, iodine, aconite, aromatic, sulphuric acid, salt and water, soap-suds, and all other kind of suds, into the face and eyes of these infinitesimal laborers, causing them to yell "calf, rope," and fall dack, blind and insensible, into what at that moment would appear to them, "hell on fire." Soon re covering however, they return, and find that the "sluice" has carried away with it a part of the wall just built; with renewed energy they commence work again, but are soon interrupted as before; in the meantime, however, they are gaining in their structure, and after months, perhaps, of toil and vexation they have accomplished their work, the abscess is crowed out, the fistulous track is obliterated, and the workmen pack their tools, after putting up the wires, return home and make a report of success. The Dentist has by this time filled the tooth, and announced to his patient, in all the "wisdom of Solomon." "I have done this; it is perfectly wonderful, madam, what a beneficial effect these remedies have had in your case."

What poor deluded, ungenerous mortals we are. If, on the first injection of creosote, the tooth had been filled, the cause of all outside trouble would have ended, and nature's forces could have, in a few days, repaired all damage.

I beg your pardon for this extended trespass on your valuable space, and hoping you all success, and that we may soon hear from "another county."—*Southern Dental Journal.*

---

